# Supplementary figures and images for: The pneumococcal two-component system SirRH is linked to enhanced intracellular survival of Streptococcus pneumoniae in influenza-infected pulmonary cells
Source: PLoS Pathog. 2020 Aug 13;16(8):e1008761. doi: 10.1371/journal.ppat.1008761 (PMC7447016; doi:10.1371/journal.ppat.1008761)

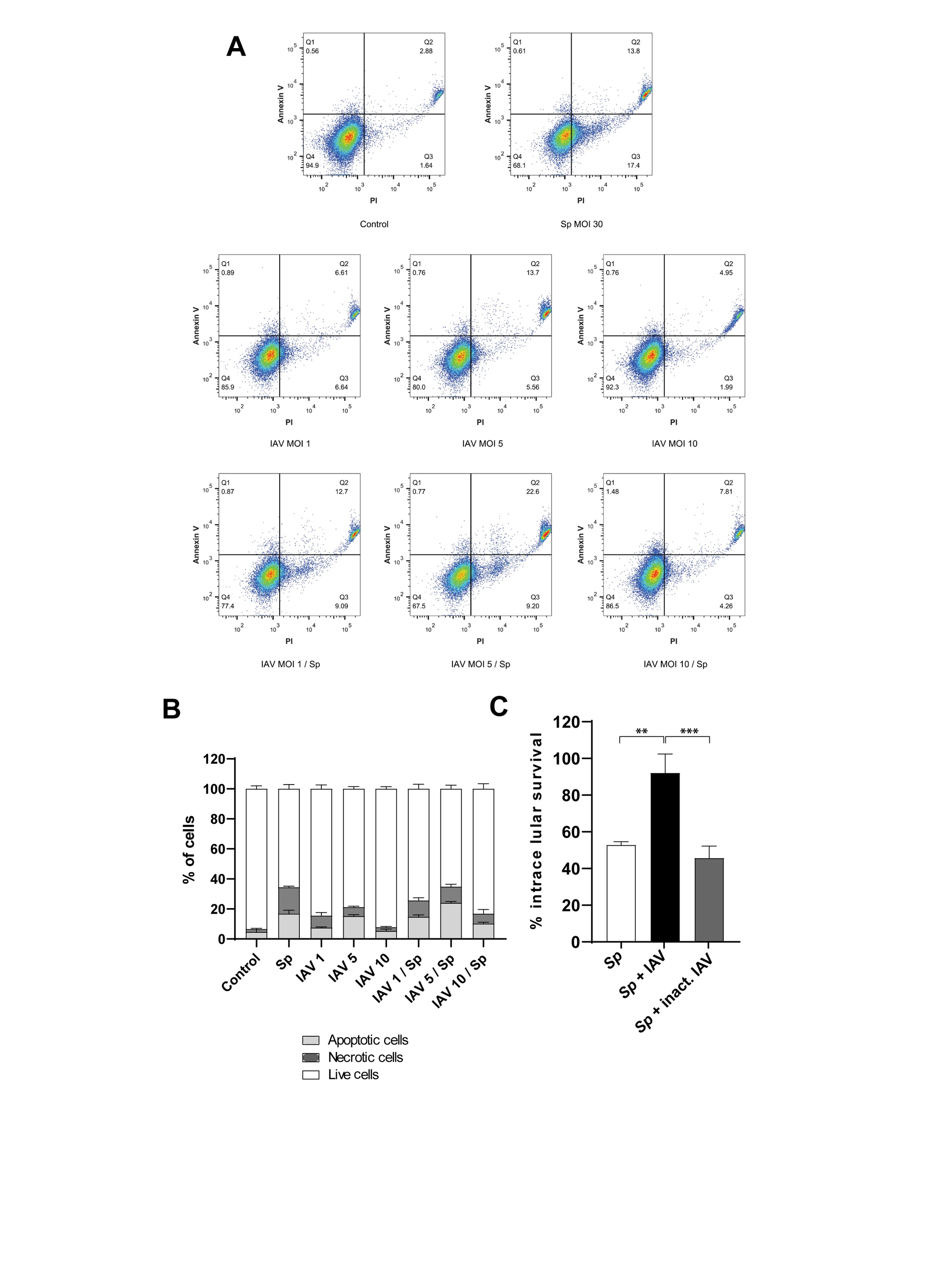

Supplement: S1 Fig — (A) A549 cells were infected with different MOI of IAV for 24 h and coinfected with a bacterial MOI of 30. Apoptosis/necrosis was measured at the single-cell level by labeling cells with annexin-V-APC and counterstaining with propidium iodide (PI). Representative data are shown and percentage of cells are indicated in each quadrant (lower left: APC-/PI-, intact cells; lower right: APC+/PI, apoptotic cells; upper left: APC-/PI+, necrotic cells; upper right: APC+/PI+, late apoptotic or necrotic cells). (B) The bar chart describes the percentage distribution of necrotic, apoptotic and viable cells after infection with different MOI of IAV or with superinfection with S. pneumoniae. (C) A549 cells were superinfected with a viral MOI of 10 using either heat-inactivated (at 56°C for 30 min) or active virus particles, and the percentage of intracellular bacterial survival was performed as described in Fig 1. (TIF) [file ppat.1008761.s001.tif]

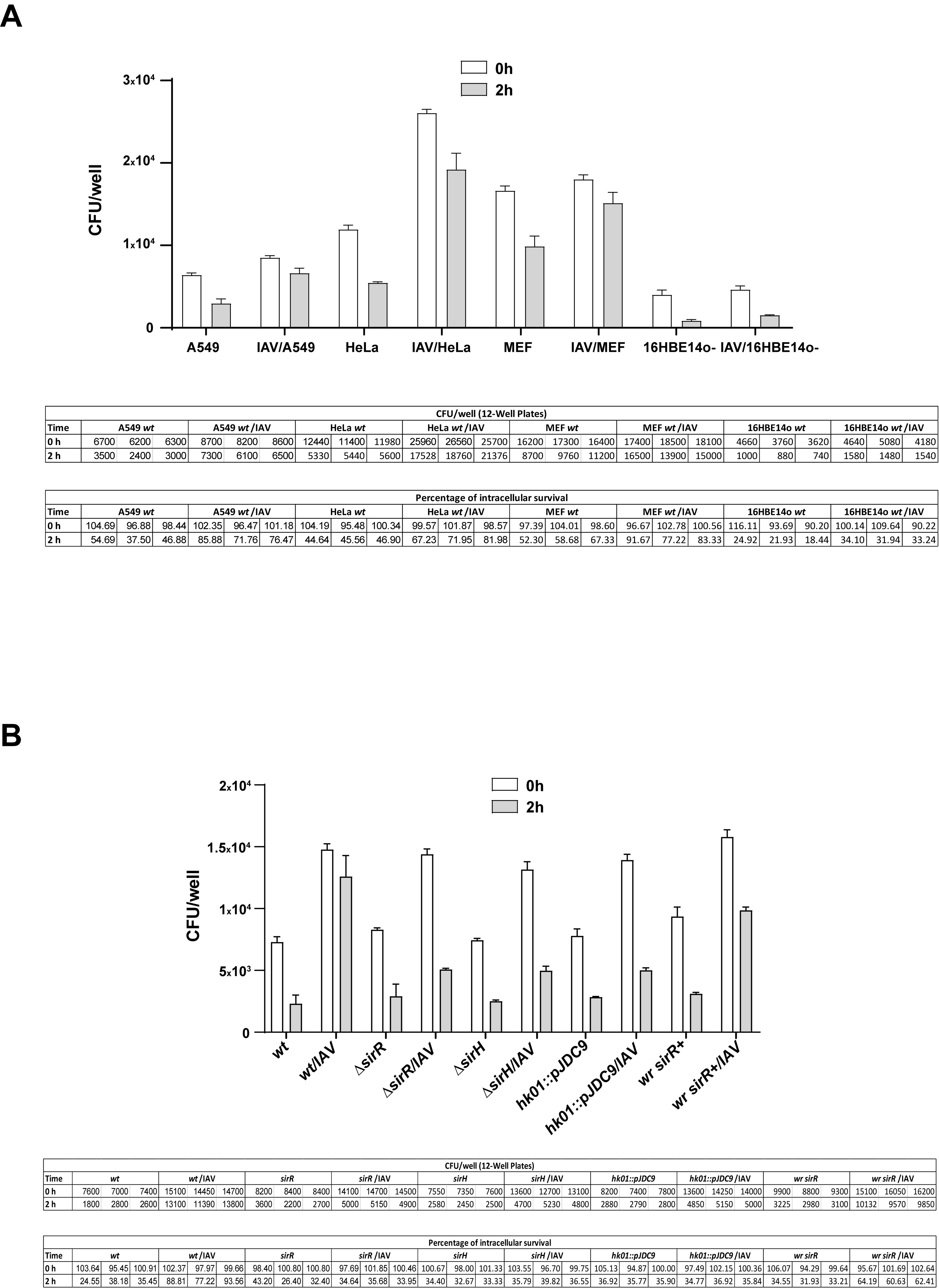

Supplement: S2 Fig — Raw data corresponding to the superinfection assays shown in Fig 1A, and for the superinfection assays in A549 cells using the pneumococcal wt, ΔsirR, ΔsirR, hk01::pJDC9 and wr sirR+ strains in Fig 1B. (A) We represented CFU counting per well at time 0h and 2h corresponding to the superinfection assays of A549, HeLa, MEF and 16HBE14o- cells shown in Fig 1A. White bars correspond to CFU at 0h and grey bars to CFU to 2h. Values represent mean ± SD. In the upper-table are represented the values of all replicates of each sample. In the bottom-table are represented the percentages of survival of all replicates of each sample. (B) We represented CFU counting per well at time 0h and 2h corresponding to the superinfection assays in A549 cells using the pneumococcal wt, ΔsirR, ΔsirR, hk01::pJDC9 and wr sirR+ strains shown in Fig 1B. White bars correspond to CFU at 0h and grey bars to CFU to 2h. Values represent mean ± SD. In the upper-table are represented the values of all replicates of each sample. In the bottom-table are represented the percentages of survival of all replicates of each sample. (TIF) [file ppat.1008761.s002.tif]

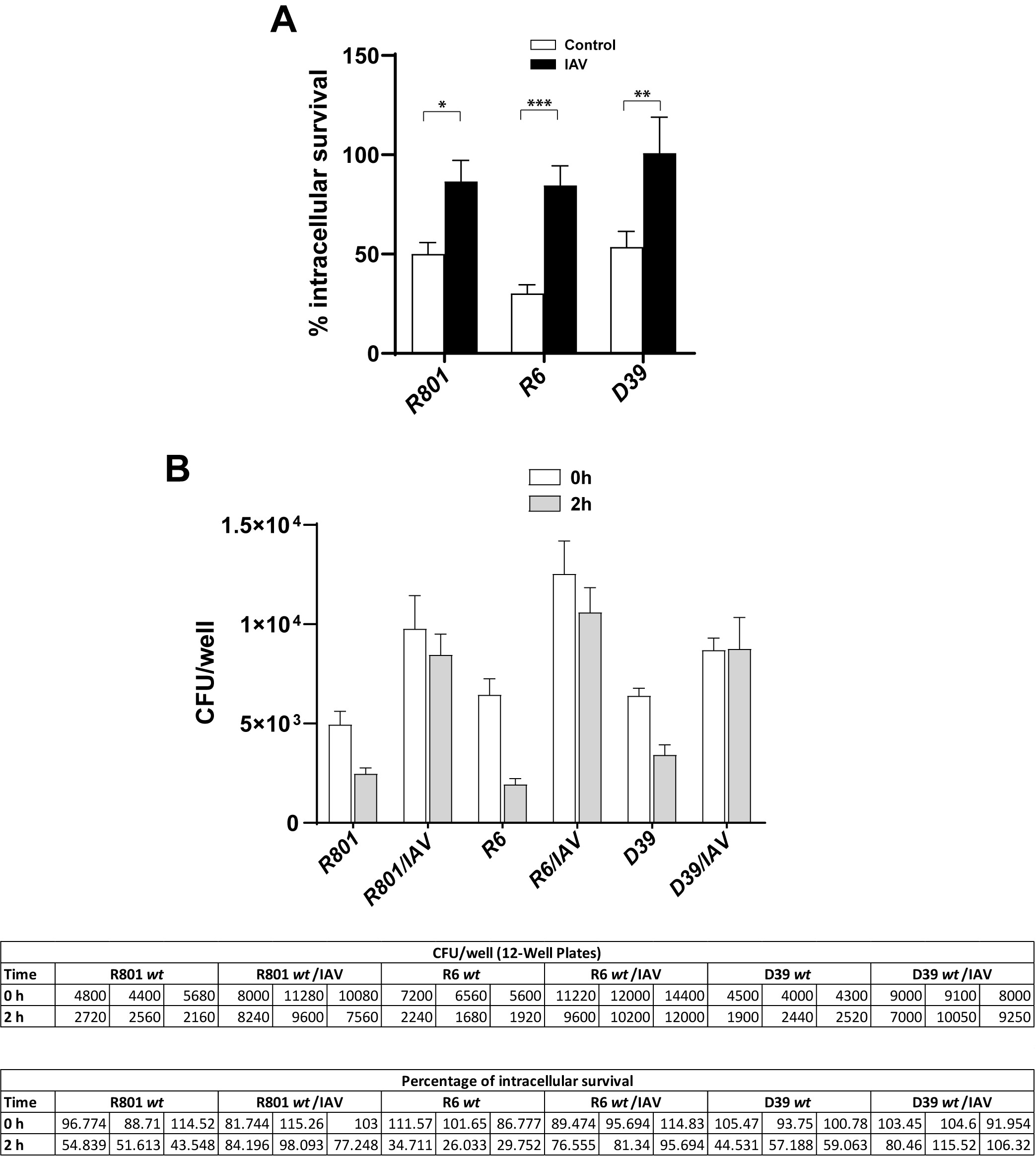

Supplement: S3 Fig — (A) A549 cells were superinfected with IAV and the pneumococcal R801, R6 and D39 cpsB strains. White bars correspond to pneumococci-infected cells and black bars to superinfected cells. (B) The raw data presented in this panel correspond to the assays shown in panel A. We represented CFU counting per well at time 0 h and 2 h, white bars correspond to CFU at 0h and grey bars to CFU to 2h. Values represent mean ± SD. In the upper-table are represented the values of all replicates of each sample. In the bottom-table are represented the percentages of survival of all replicates of each sample. (TIF) [file ppat.1008761.s003.tif]

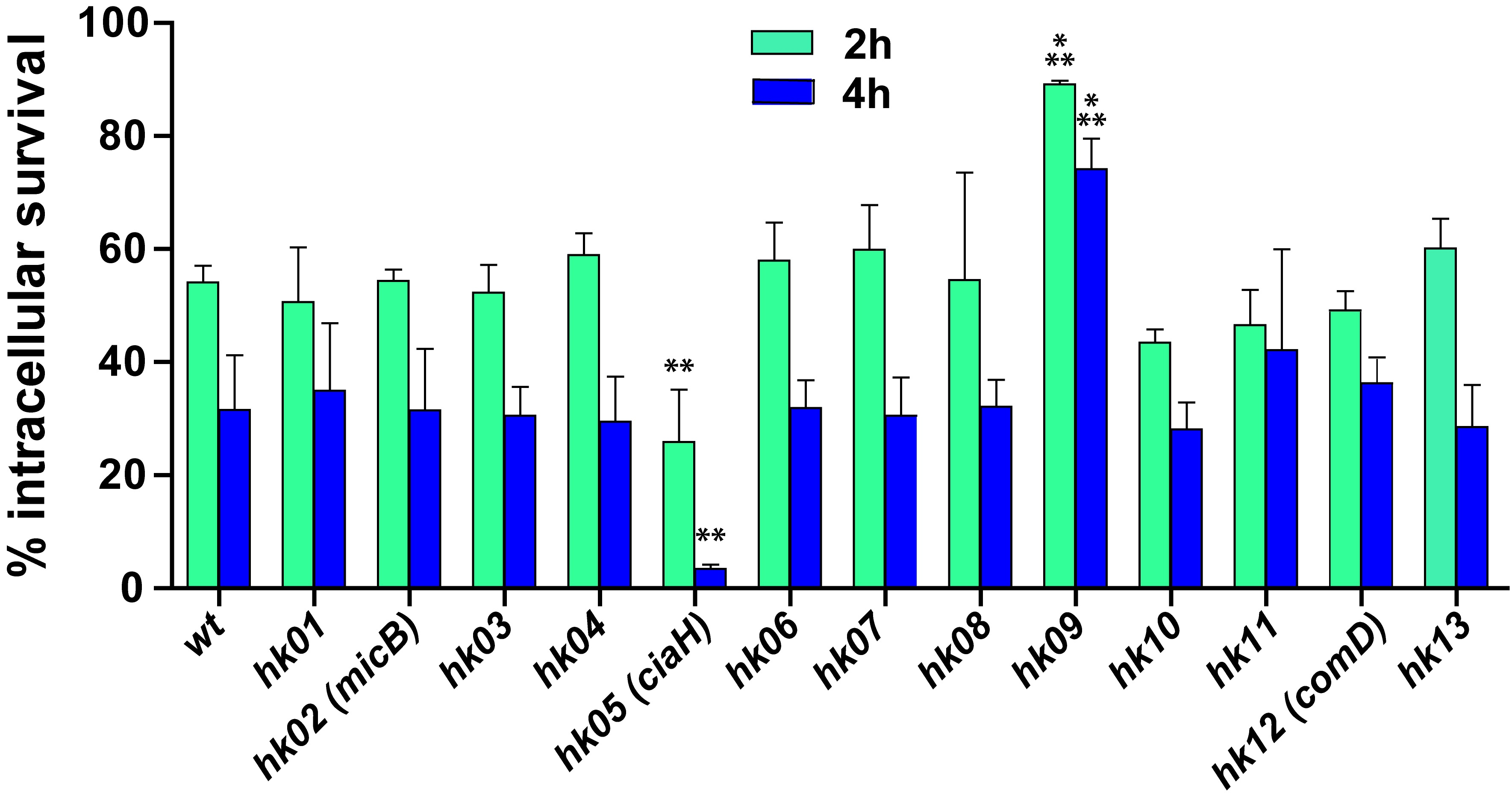

Supplement: S4 Fig — A549 cells were infected with different hk mutants and its intracellular survival capacity was determined as described for non-virus infected cells in the Fig 1 legend, and these results were compared with those obtained for the wt strain. Green bars and blue bars correspond to 2 h and 4 h of incubation after antibiotic treatment, respectively. (TIF) [file ppat.1008761.s004.tif]

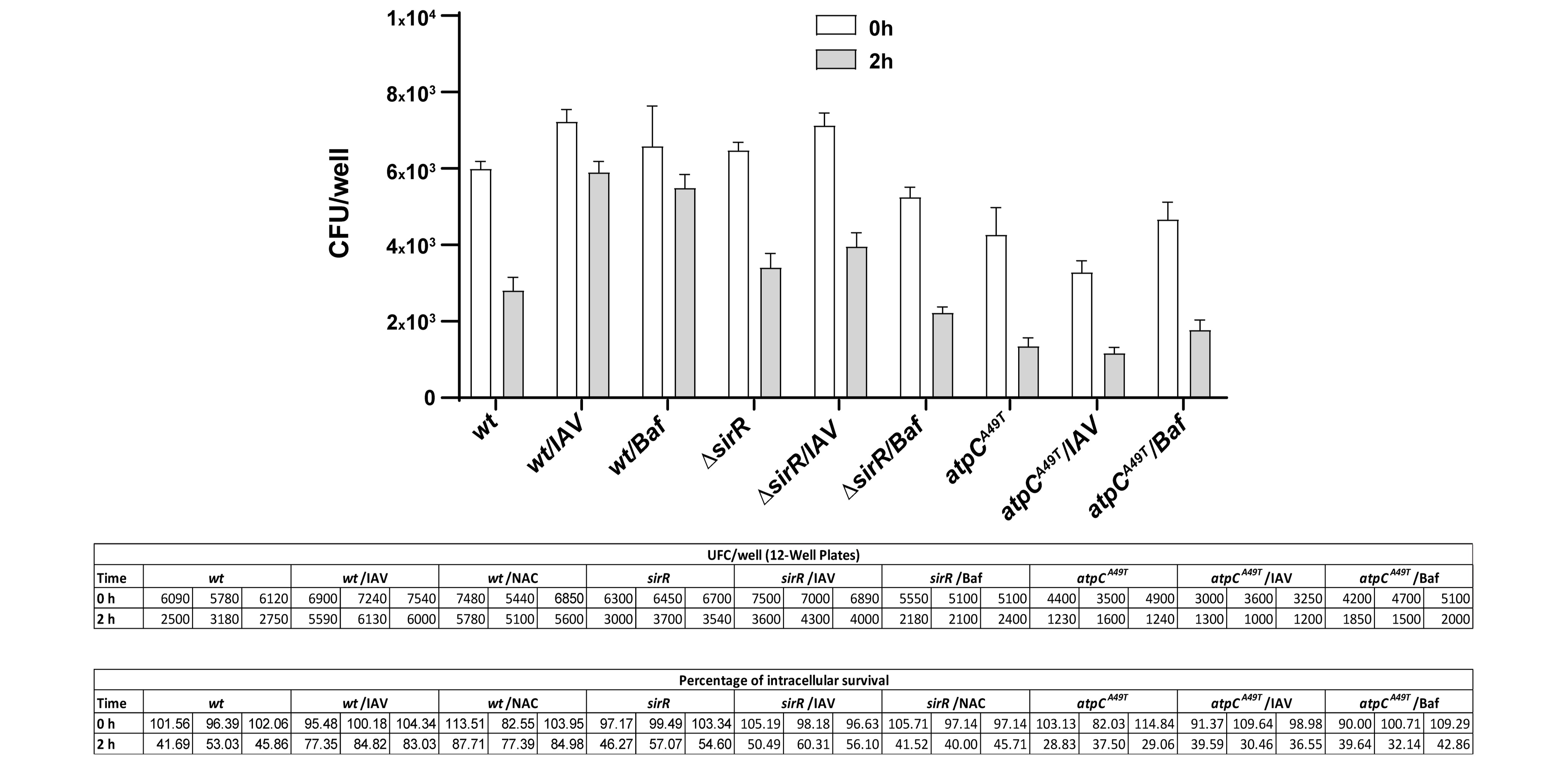

Supplement: S5 Fig — Raw data corresponding to the intracellular survival of the wt, ΔsirR, ΔatpCA49T strains in A549 cells shown in Fig 2B, which were either infected with IAV or treated with 100 nM Bafilomycin A1, and compared with non-treated A549 cells. We represented CFU counting per well at time 0 h and 2 h, white bars correspond to CFU at 0 h and gray bars to CFU to 2h. Values represent mean ± SD. In the upper-table are represented the values of all replicates of each sample. In the bottom-table are represented the values of all replicates of each sample. (TIF) [file ppat.1008761.s005.tif]

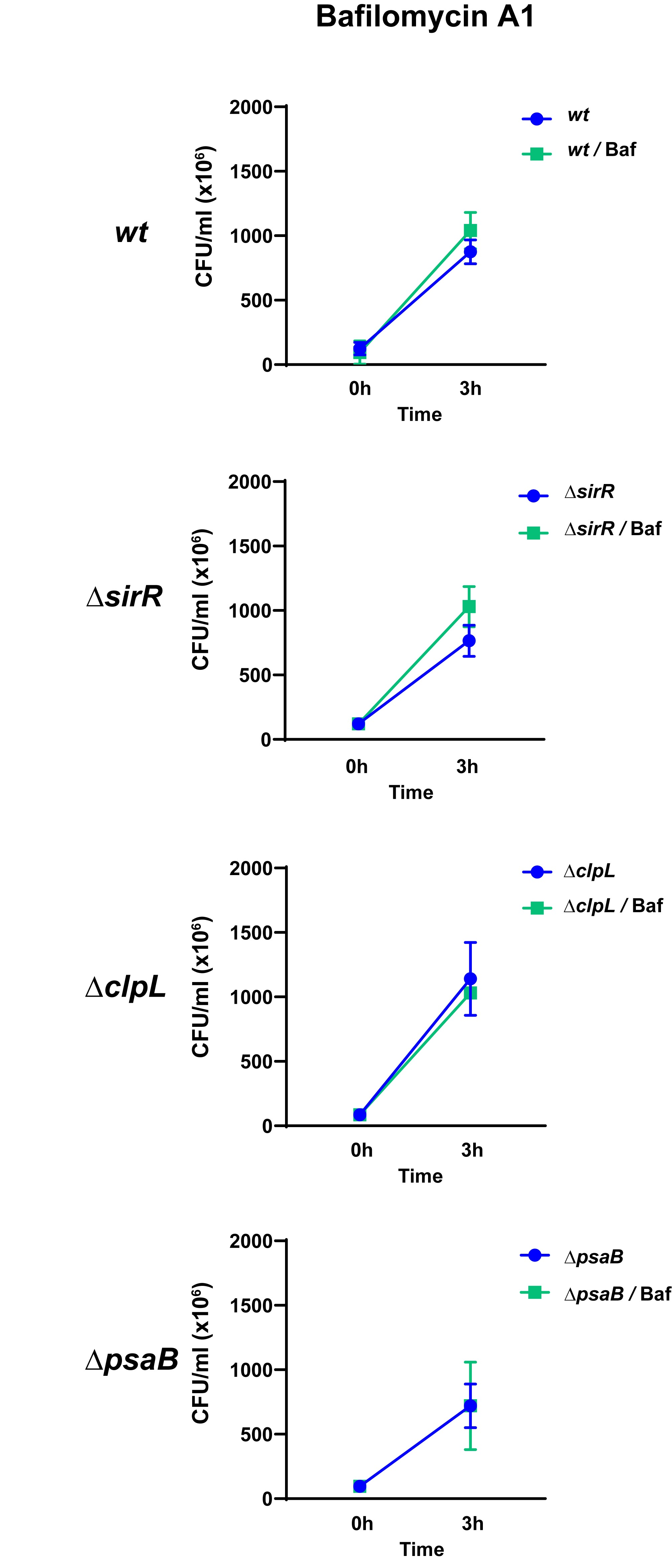

Supplement: S6 Fig — The wt, ΔsirR, ΔclpL, and ΔpsaB strains were grown at 37°C for 3 h in both BHI and BHI containing 100 nM Bafilomycin A1 (100nM). We counted CFU at 0h and 3h post-treatment. For these strains, we observed no significant difference in their growth curves when bacteria cells were treated with Bafilomycin A1 and compared with non-treated pneumococci. The blue lines and circles correspond to control, and green lines and squares correspond to Bafilomycin A1-treatment conditions. Values represent the mean ± SD. Statistical significance was calculated by Student’s t-test. (TIF) [file ppat.1008761.s006.tif]

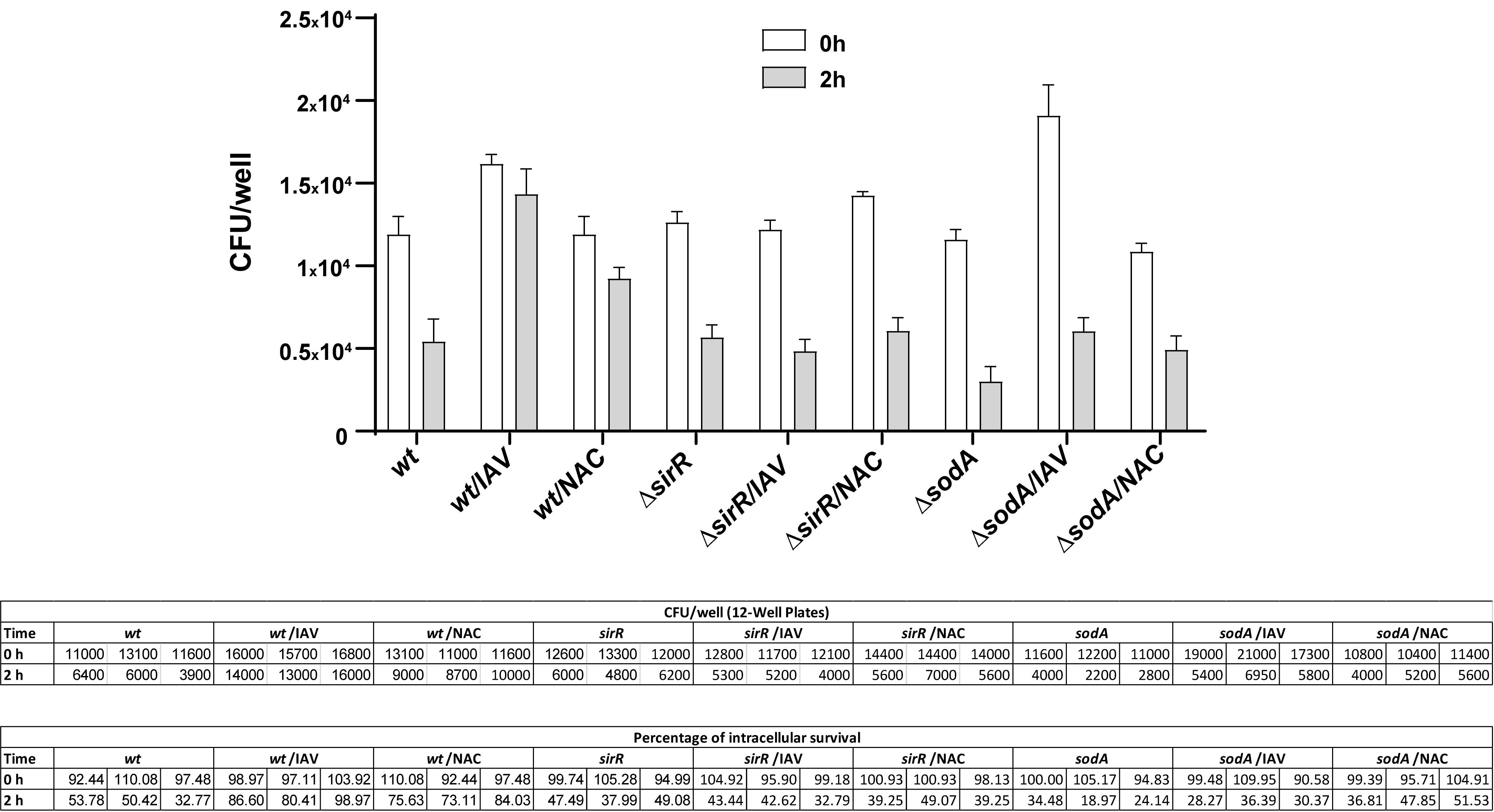

Supplement: S7 Fig — Raw data corresponding to the intracellular survival of the ΔsirR, ΔsodA and wt strains in A549 cells shown in Fig 3B, which were either infected with IAV or treated with 5 mM NAC. We represented CFU counting per well at time 0 h and 2 h, white bars correspond to CFU at 0h and green bars to CFU to 2h. Values represent mean ± SD. In the upper-table are represented the values of all replicates of each sample. In the bottom-table are represented the percentages of survival of all replicates of each sample. (TIF) [file ppat.1008761.s007.tif]

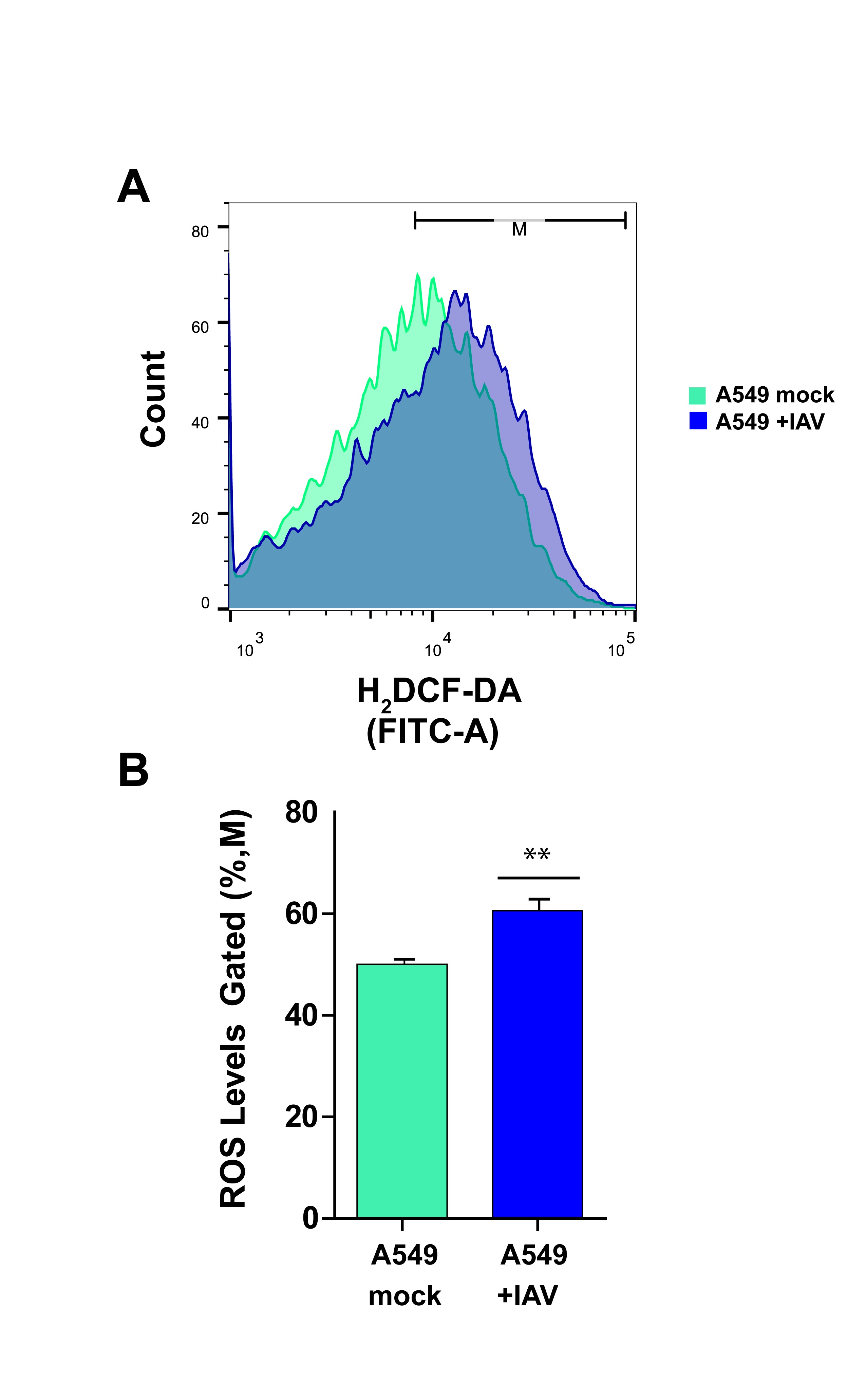

Supplement: S8 Fig — (A) Representative flow cytometry histogram showing results of H2DCF-DA staining (a measurement of ROS levels) of IAV-infected A549 cells or mock-A549 cells. (B) Bar graph depicting results of IAV-infected A549 cells compared with non-infected cells. Data are representative of at least three independent experiments. (TIF) [file ppat.1008761.s008.tif]

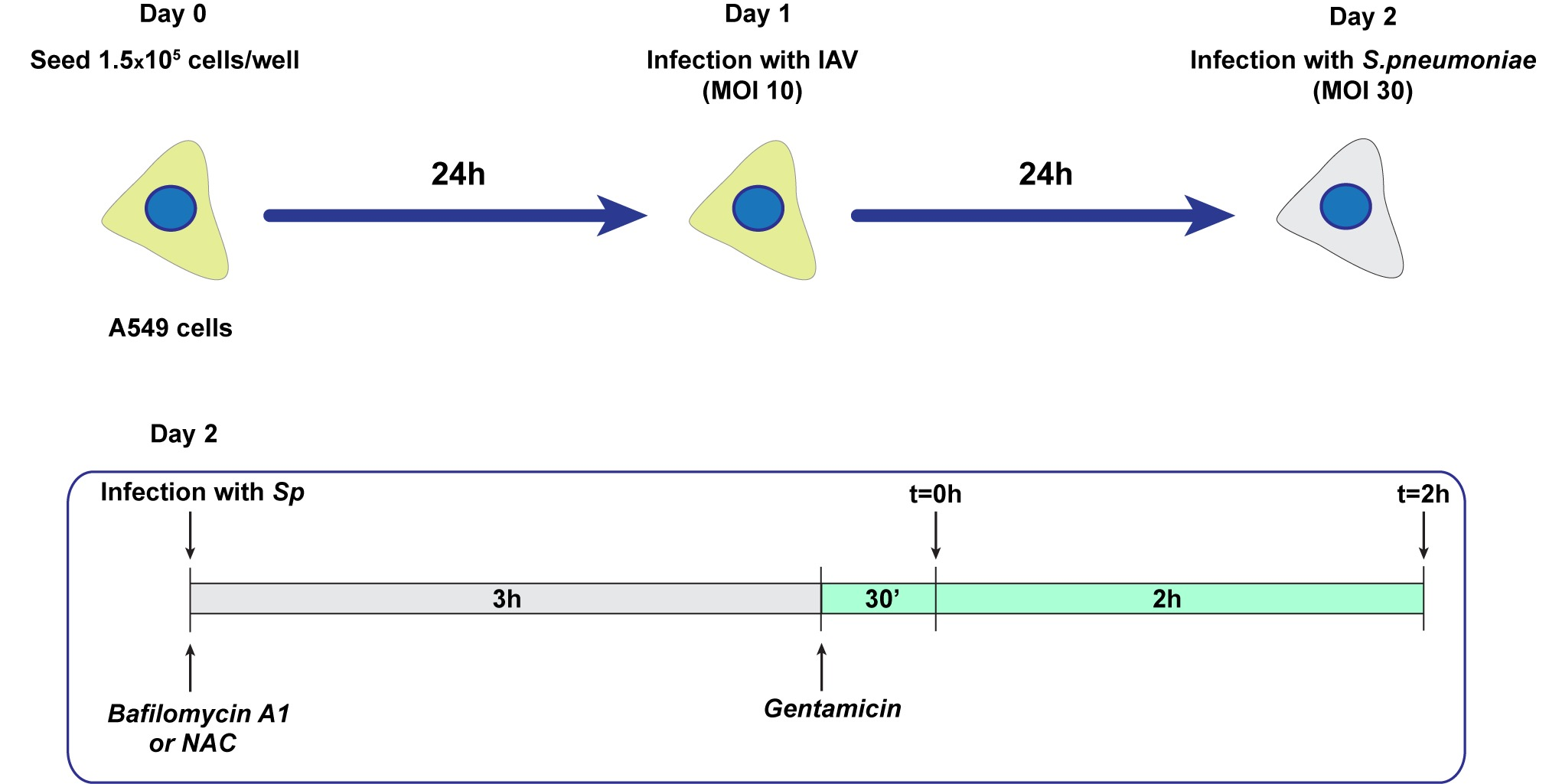

Supplement: S9 Fig — (TIF) [file ppat.1008761.s009.tif]

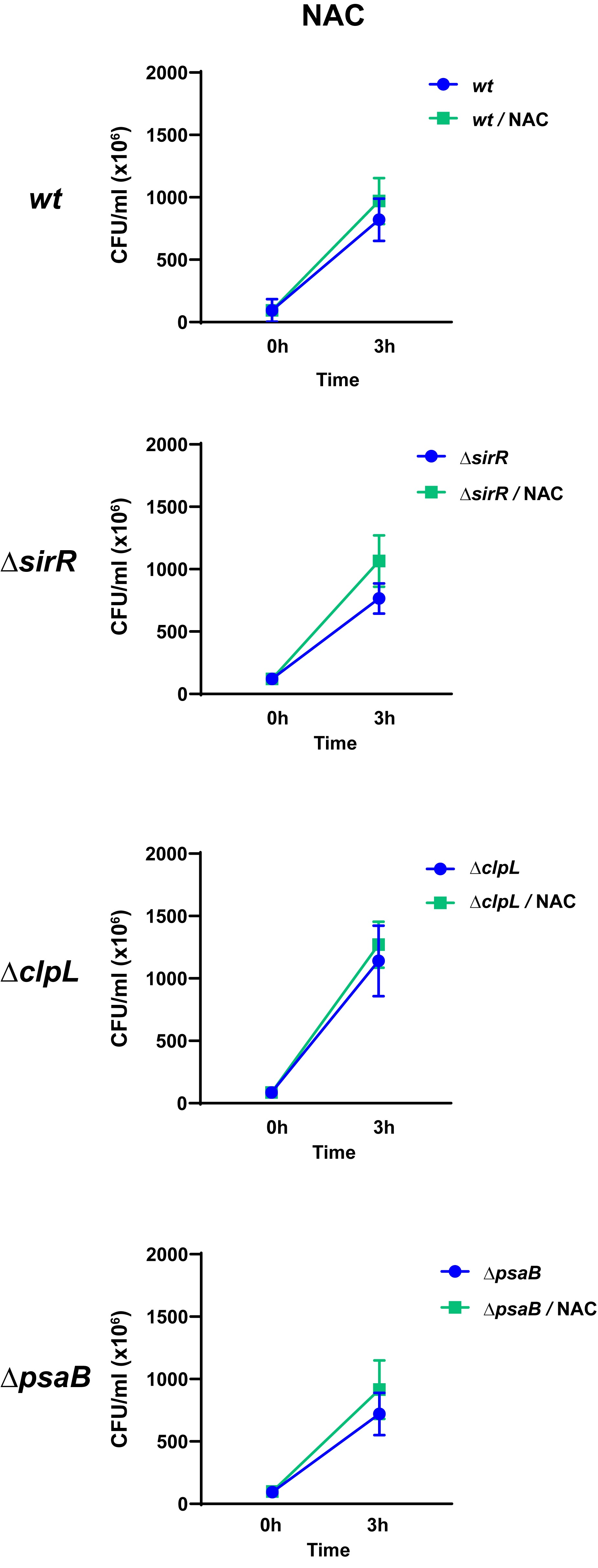

Supplement: S10 Fig — The wt, ΔsirR, ΔclpL, and ΔpsaB strains were grown at 37°C for 3 h in both BHI and BHI containing 5 mM N-acetyl-L-cysteine (NAC). We counted CFU at 0h and 3h post-treatment. For all the strains, we observed no significant difference in growth curves between control and NAC-treatment condition. The blue lines and circles correspond to control, and green lines and squares correspond to NAC-treatment conditions. Values represent the mean ± SD. Statistical significance was calculated by Student´s t-test. (TIF) [file ppat.1008761.s010.tif]

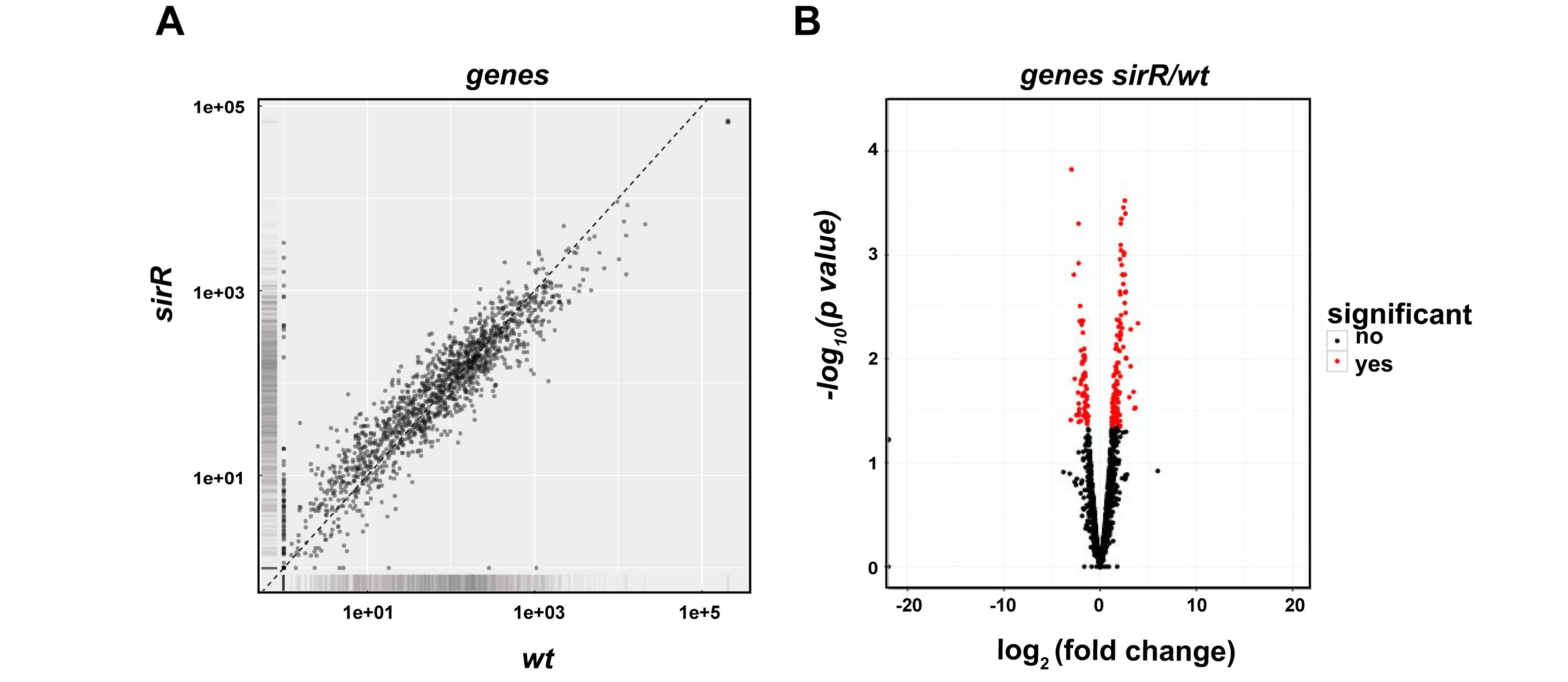

Supplement: S11 Fig — (A) Gene expression scatter plot in samples obtained from the wt strain and the ΔsirR mutants, with the x-axis representing the gene expression values for the control condition (wt) and the y-axis representing those for the treated condition (ΔsirR). Each black dot represents a significant single transcript, with the vertical position of each gene representing its expression level in the experimental conditions and the horizontal one representing its control strength. Thus, genes that fall above the diagonal are over-expressed whereas genes that fall below the diagonal are under-expressed as compared to their median expression levels in the experimental groups. (B) Volcano plot of gene expression in wt vs ΔsirR samples measured by RNAseq. The y-axis represents the mean expression value of the log10 (p-value), while the x-axis displays the log2 fold change value. Black dots represent genes with an expression 2-fold higher in the ΔsirR mutant relative to strain wt with a p-value < 0.05, with red dots signifying genes with an expression 2-fold lower in the ΔsirR mutant, which are relative to strain wt with a p < 0.05. (TIF) [file ppat.1008761.s011.tif]

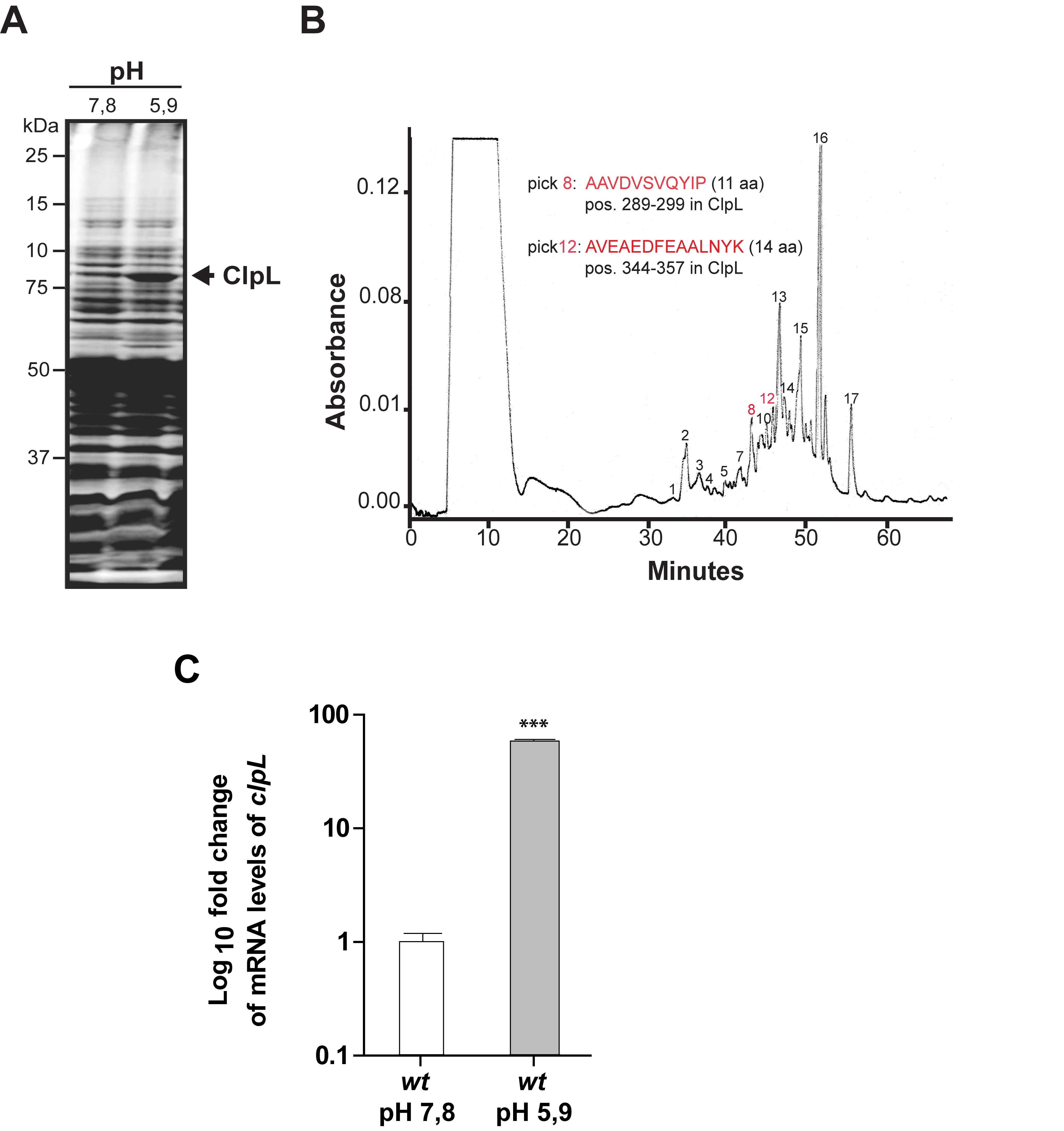

Supplement: S12 Fig — (A) SDS-PAGE analysis of protein extracts obtained from the wt cells grew at slightly alkaline (pH 7.8) or acidic (pH 5.9) culture media. The protein band subjected to N-terminal sequencing is indicated by an arrow. (B) The N-terminal sequence obtained by Edman degradation were analyzed by tryptic digestion and HLPC-protein sequencer. The m/z values of ions matching peptides derived from the 78-kDa protein band are indicated by numbers. The amino acids sequences corresponding to pick 8 (11 amino acids) and pick 12 (14 amino acids) corresponded to the ClpL chaperone, according to the R6 pneumococcal genome (https://www.uniprot.org/ proteomes/UP000000586). (C) Transcription levels of the clpL gene increased in cells exposed to acidic pH. The wt cells were grown in BHI/pH 7.8 to the mid-exponential phase and resuspended in ABM/pH 5.9, and total RNA was extracted at 1 h. The fold change in gene expression was measured by quantitative real-time PCR and calculated using the 2–ΔΔCT method. The gyrA gene was used as the internal control. Error bars indicate the standard deviation of the mean. INSTAT software was used to perform Dunnet’s statistical comparison test for each strain. References: **p< 0.01; ***p< 0.001. (TIF) [file ppat.1008761.s012.tif]

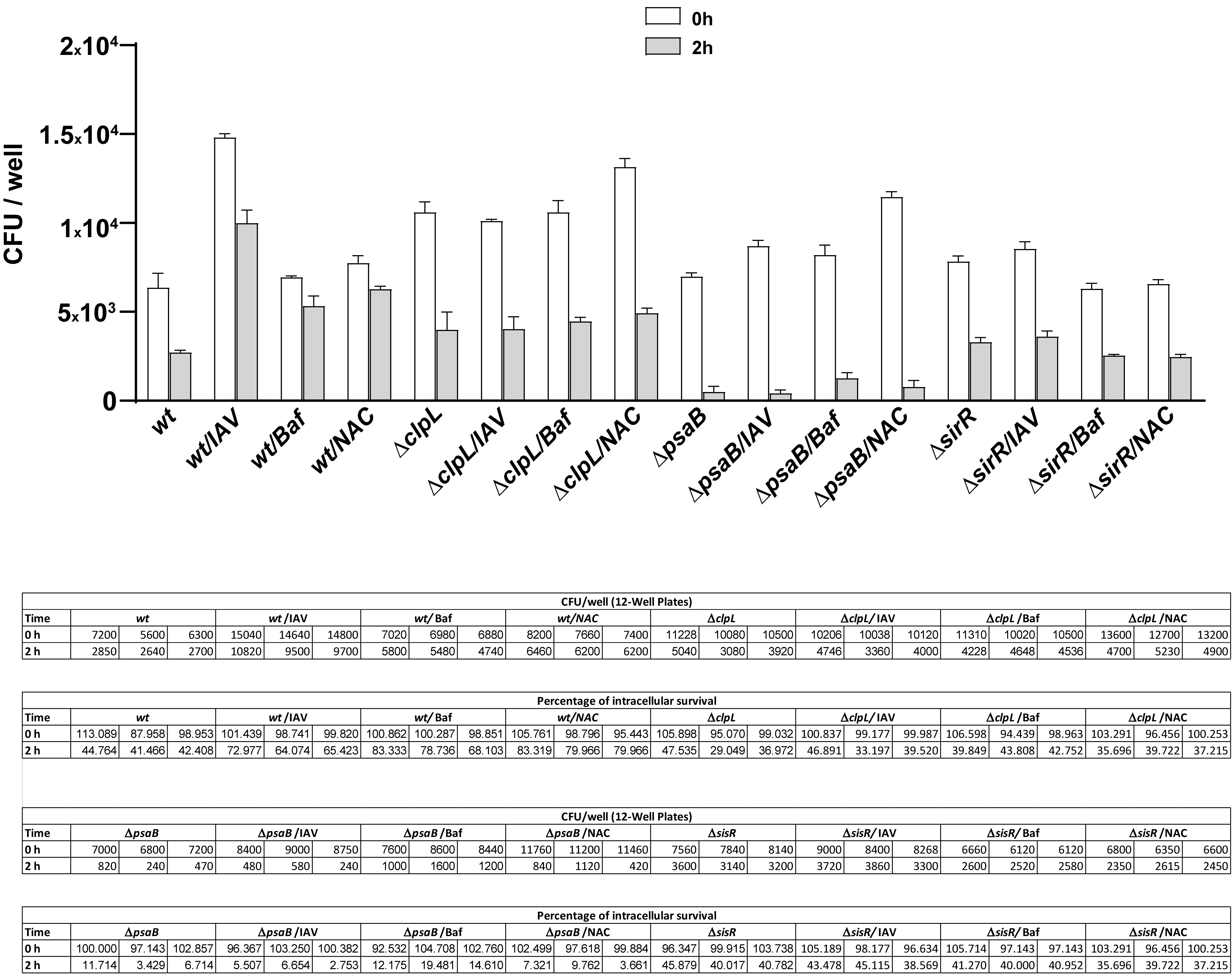

Supplement: S13 Fig — (A) Raw data corresponding to the intracellular survival assays shown in Fig 6E for the wt, ΔclpL, ΔpsaB and ΔsirR strains, with either bacterial infection, IAV/bacterial coinfection, or bacterial infection with a 100 nM Bafilomycin A1 treatment. We represented CFU counting per well at time 0 h and 2 h, white bars correspond to CFU at 0h and grey bars to CFU to 2 h. Values represent mean ± SD. In the upper-table are represented the values of all replicates of each sample. In the bottom-table are represented the percentages of survival of all replicates of each sample. (B) Raw data corresponding to the intracellular survival assays for the wt, ΔclpL, ΔpsaB and ΔsirR strains, with either bacterial infection, IAV/bacterial coinfection, or bacterial infection with a 5 mM NAC treatment shown in Fig 6E. We represented CFU counting per well at time 0 h and 2 h, white bars correspond to CFU at 0h and grey bars to CFU to 2h. Values represent mean ± SD. In the upper-table are represented the values of all replicates of each sample. In the bottom-table are represented the percentages of survival of all replicates of each sample. (TIF) [file ppat.1008761.s013.tif]

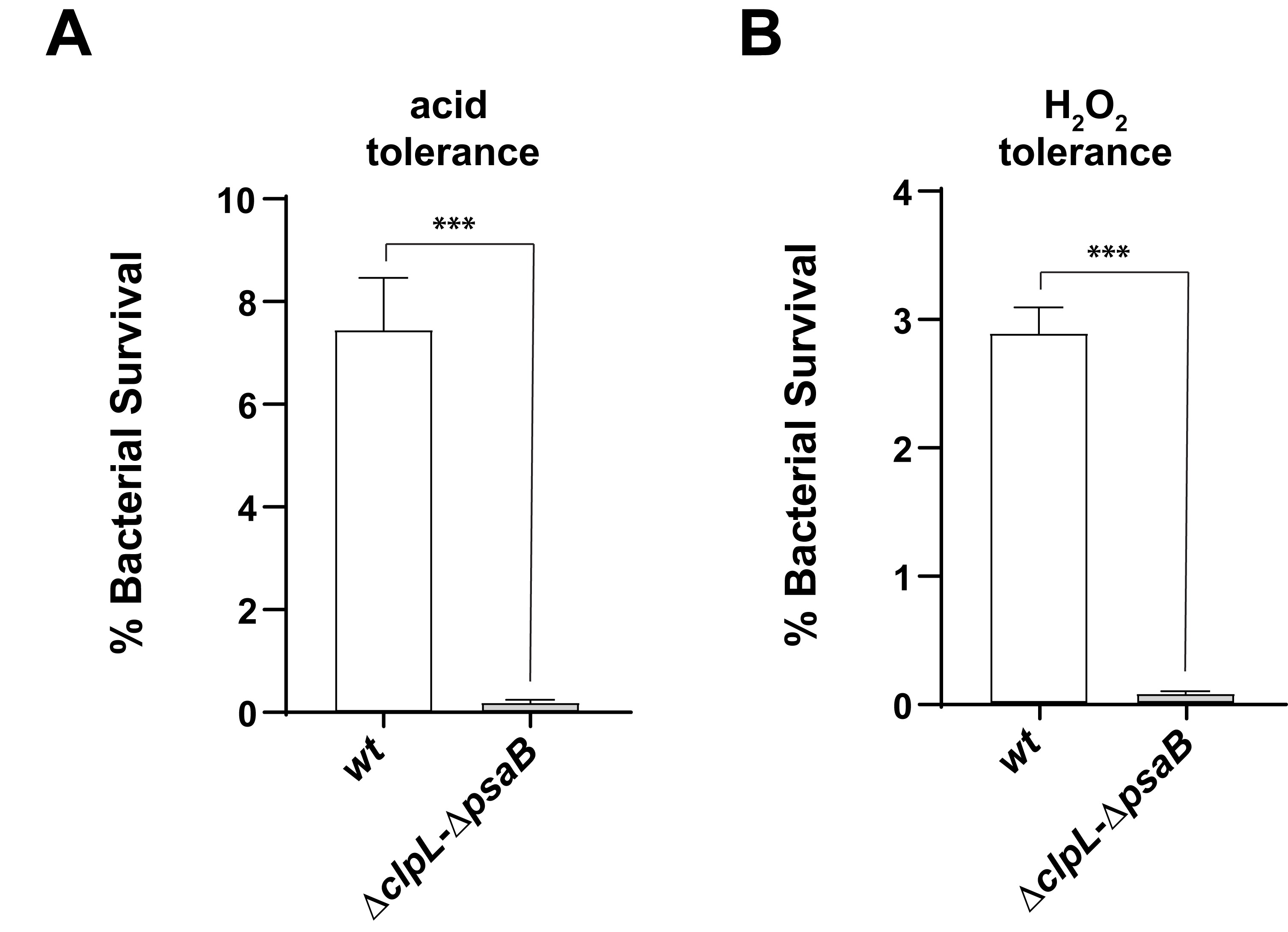

Supplement: S14 Fig — (A) The wt and ΔclpL-ΔpsaB cells were grown in BHI at 37°C until an OD620nm 0.3, and then incubated in ABM medium at pH 4.8 for 1 h at 37°C. Viable cells were assessed by spreading dilutions in BHI-blood-agar plates and incubating these at 37°C for 16 h. (B) The wt and ΔclpL-ΔpsaB cells were grown in BHI and then exposed at BHI medium containing 20 mM H2O2 for 2 h. After that, viable cells were determined by spreading dilutions in BHI-blood-agar plates and incubating these at 37°C for 16 h. Data are representative of at least three independent experiments. Values represent mean ± SD Statistical significance was calculated by Student’s t-test, p<0.001 (***). (TIF) [file ppat.1008761.s014.tif]

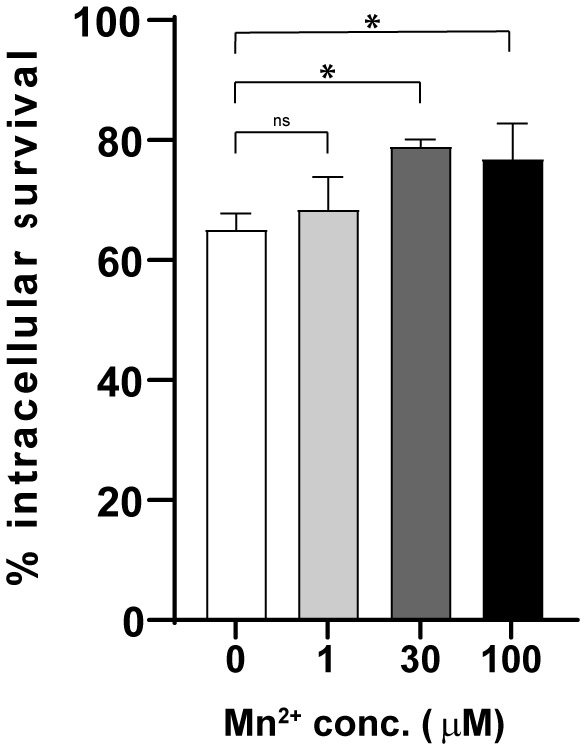

Supplement: S15 Fig — The wt strain was cultivated in BHI with different concentrations of MnSO4 (1, 30 and 100 μM) until reaching an OD600nm 0.3. Then, A549 cells were infected with these Mn-pretreated bacterial cells, and the intracellular survival for each condition was measured. White bar corresponds to control and the shades of gray correspond to different concentrations of Mn2+. One representative experiment of three independent assays is shown. Values represent the mean ± SD. Statistical significance was calculated by one-way ANOVA, followed by Post Hoc Test Tukey, p<0.05 (*). (TIF) [file ppat.1008761.s015.tif]

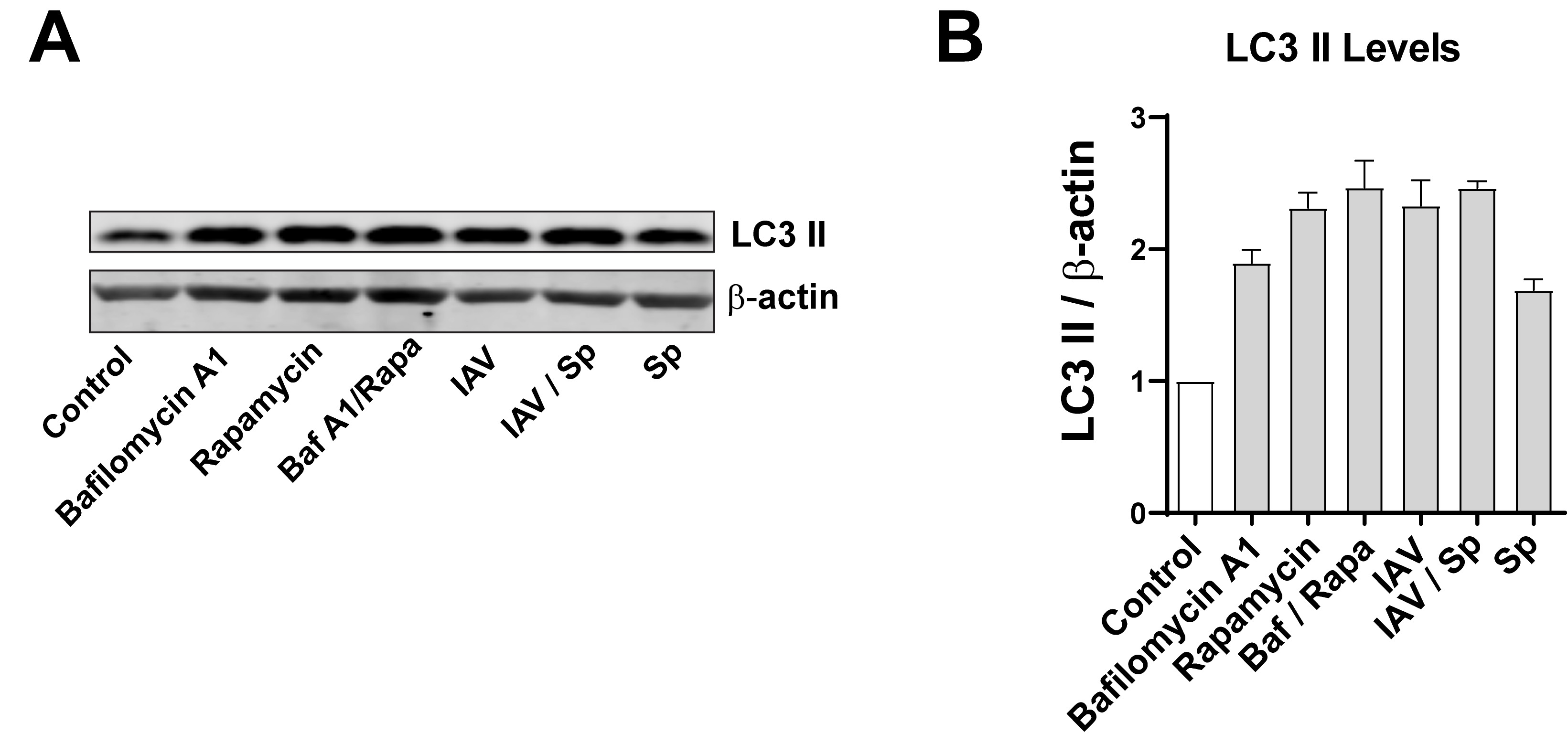

Supplement: S16 Fig — (A) LC3-II levels are induced by superinfection with IAV and S. pneumoniae. A549 cells were infected with IAV (MOI 10), S. pneumoniae (MOI 30) and coinfected as described in the Fig 1A legend. As controls, A549 cells were also treated with inducers (Rapamycin) and inhibitors (Bafilomycin A1) of the autophagy process. Cell lysates were subjected to Western blot analysis using anti-LC3-II, anti-beta-actin antibodies with data being representative of at least three independent experiments. (B) Quantification of the LC3-II level in western blot: bar graphs represent LC3-II relative intensity (LC3-II/β-actin) with data being representative of at least three independent experiments. (TIF) [file ppat.1008761.s016.tif]

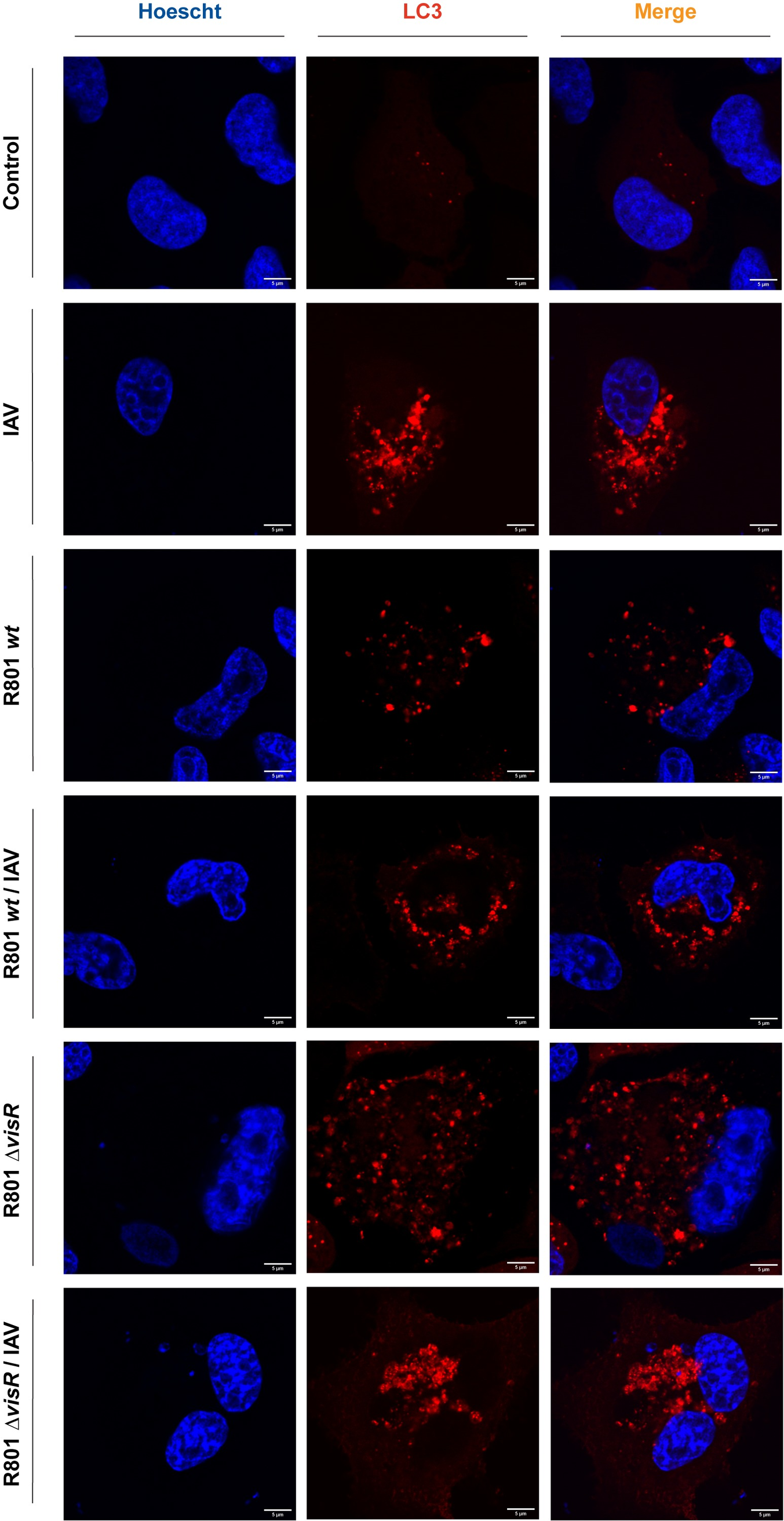

Supplement: S17 Fig — The A549 cells were transfected with the mKate2-hLC3 plasmids for 24 hours, and followed by either IAV, individual pneumococcal strains (wt or ΔsirR), or superinfection with each strain. The far-red (mKate2) fluorescence in the cells was monitored using an Olympus FluoView FV1000 confocal laser scanning microscope. (TIF) [file ppat.1008761.s017.tif]

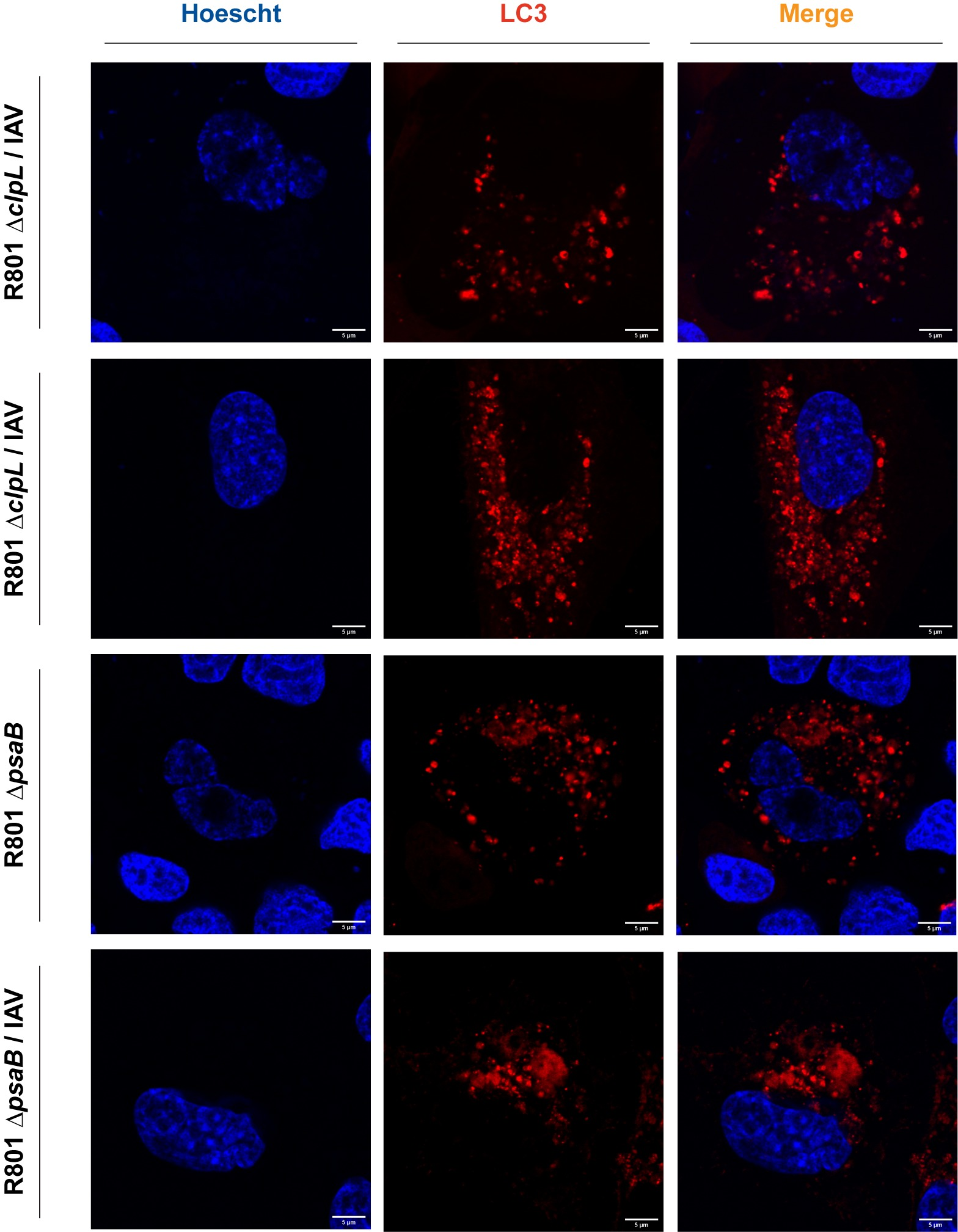

Supplement: S18 Fig — The A549 cells were transfected with the mKate2-hLC3 plasmids for 24 hours, and followed by either IAV, individual pneumococcal strains (ΔclpL or ΔpsaB), or superinfection with each strain. These assays were processed at the same time that those shown in the S17 Fig. The far-red (mKate2) fluorescence in the cells was monitored using an Olympus FluoView FV1000 confocal laser scanning microscope. (TIF) [file ppat.1008761.s018.tif]

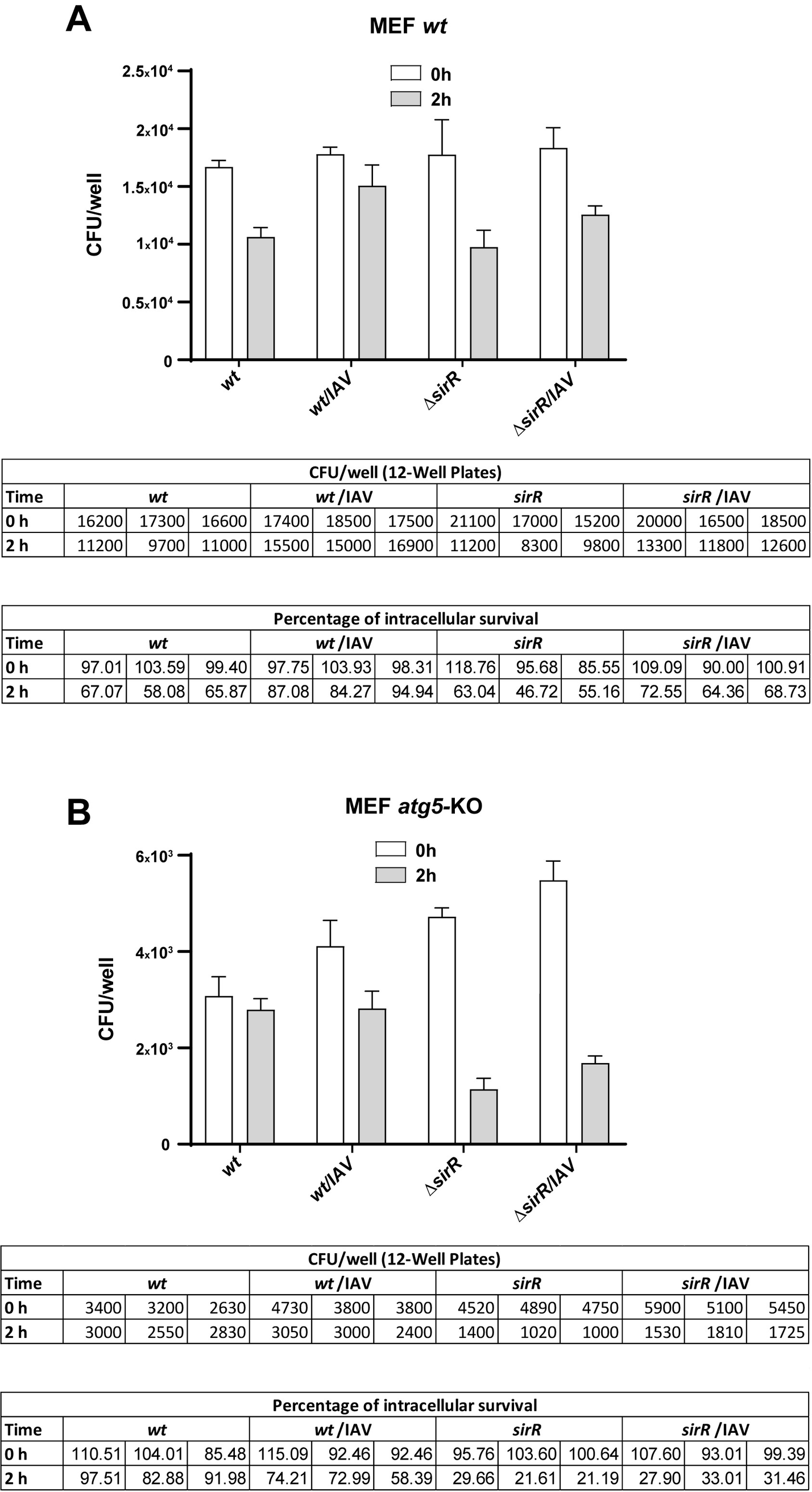

Supplement: S19 Fig — Raw data corresponding to the intracellular survival assays shown in Fig 7A and 7B for the MEF wt (A) and MEF atg5-KO cells (B), with either bacterial infection or IAV/bacterial coinfection. We represented CFU counting per well at time 0 h and 2 h, white bars correspond to CFU at 0h and grey bars to CFU to 2 h. Values represent mean ± SD. In the upper-table are represented the values of all replicates of each sample. In the bottom-table are represented the percentages of survival of all replicates of each sample. (TIF) [file ppat.1008761.s019.tif]
